# DNA Methylation in Pediatric Obstructive Sleep Apnea: An Overview of Preliminary Findings

**DOI:** 10.3389/fped.2018.00154

**Published:** 2018-05-29

**Authors:** Evanthia Perikleous, Paschalis Steiropoulos, Argyris Tzouvelekis, Evangelia Nena, Maria Koffa, Emmanouil Paraskakis

**Affiliations:** ^1^MSc Program in Sleep Medicine, Medical School, Democritus University of Thrace, Alexandroupolis, Greece; ^2^Division of Immunology, Biomedical Sciences Research Center “Alexander Fleming”, Athens, Greece; ^3^Laboratory of Hygiene and Environmental Protection, Medical School, Democritus University of Thrace, Alexandroupolis, Greece; ^4^Department of Molecular Biology and Genetics, Democritus University of Thrace, Alexandroupolis, Greece; ^5^Department of Pediatrics, Medical School, Democritus University of Thrace, Alexandroupolis, Greece

**Keywords:** obstructive sleep apnea, DNA methylation, Forkhead box P3, endothelial Nitric Oxide Synthase gene, intermittent hypoxia

## Abstract

Obstructive sleep apnea (OSA) is characterized by phenotypic variations, which can be partly attributed to specific gene polymorphisms. Recent studies have focused on the role of epigenetic mechanisms in order to permit a more precise perception about clinical phenotyping and targeted therapies. The aim of this review was to synthesize the current state of knowledge on the relation between DNA methylation patterns and the development of pediatric OSA, in light of the apparent limited literature in the field. We performed an electronic search in PubMed, EMBASE, and Google Scholar databases, including all types of articles written in English until January 2017. Literature was apparently scarce; only 2 studies on pediatric populations and 3 animal studies were identified. Forkhead Box P3 (FOXP3) DNA methylation levels were associated with inflammatory biomarkers and serum lipids. Hypermethylation of the core promoter region of endothelial Nitric Oxide Synthase (eNOS) gene in OSA children were related with decreased eNOS expression. Additionally, increased expression of genes encoding pro-oxidant enzymes and decreased expression of genes encoding anti-oxidant enzymes suggested that disturbances in oxygen homeostasis throughout neonatal period predetermined increased hypoxic sensing in adulthood. In conclusion, epigenetic modifications may be crucial in pediatric sleep disorders to enable in-depth understanding of genotype-phenotype interactions and lead to risk assessment. Epigenome-wide association studies are urgently needed to validate certain epigenetic alterations as reliable, novel biomarkers for the molecular prognosis and diagnosis of OSA patients with high risk of end-organ morbidity.

## Introduction

Obstructive sleep apnea (OSA) is characterized by repeated episodes of complete or partial blockage of the upper airway during sleep, leading to oxygen desaturation and hypercapnia, creating augmentation of respiratory effort and alterations in intrathoracic pressures, eventually climaxing in arousals. OSA is associated with considerable morbidity related to metabolic dysfunctions as well as impairment of the cardiovascular and neurocognitive systems (1). Ongoing effort to develop and implement alternative tests, such as novel diagnostic biomarkers, in terms of identifying individuals vulnerable to high risk for cardiovascular morbidity is being made (1, 2).

Over the past few decades research has concentrated on molecular mechanisms that mediate and sustain epigenetic changes such as DNA methylation, histone modifications, and non-coding RNA-associated gene silencing, including microRNAs (miRNAs) (3). These epigenetic modifications seem to influence the ability of many strains or species to react to environmental stimuli through gene variations in their gene expression. Few paradigms of environmental stimuli linked with vulnerability to OSA risk are exposure to smoking, dietary habits, fitness and air pollution. DNA methylation is a well-characterized epigenetic biochemical process that typically occurs by the covalent addition of a methyl group at the 5-carbon of the cytosine ring resulting in 5-methylcytosine, changing the appearance and structure of DNA, contrary without altering the primary DNA sequence. DNA methylation authorizes a silent chromatin stage by cooperating with proteins that reshape nucleosomes (4, 5). DNA methylation in regulatory genomic regions, such as the promoter region, can change the transcriptional state of the gene, thus altering gene expression and also the expression of gene-related products (6, 7).

The genetic research of OSA is in its infancy. Recent studies have linked epigenetic alterations, such as differences in methylation patterns, with inflammatory diseases like asthma (8). The association between the immunological principle and the manifestation of pediatric OSA has also been recently suggested (9). Epigenetic changes may represent the direct outcome of OSA, or delineate former epigenetic alterations that occurred during previous generations, gestation, or early post-natal life (9). It is possible that epigenetic modifications of certain genes lead to markedly different phenotypic variance in children with OSA (10). The premise that epigenetic alterations caused by early life events may contribute to increased risk of developing OSA has recently emerged through preclinical models (11–14).

The expeditiously evolving field of epigenetics has enlarged our perception about phenotypic fluctuation of many diseases, such as cancer, asthma, type II diabetes, cardiovascular disease, and autistic spectrum disorders (15). The main determining factors for the phenotypic trajectories in pediatric OSA remain, in a great proportion, unidentified, and the need for large population-based genome-wide studies appears imperative. Indeed, epigenetic modifications add a new determinant to the interpretation of OSA phenotypic complexity. The aim of the present review was to summarize current knowledge on the context of DNA methylation patterns in the development of pediatric OSA, in order to address some of the lesser known determinants of phenotypic diversity in pediatric OSA.

## Materials and methods

**Figure 1:**
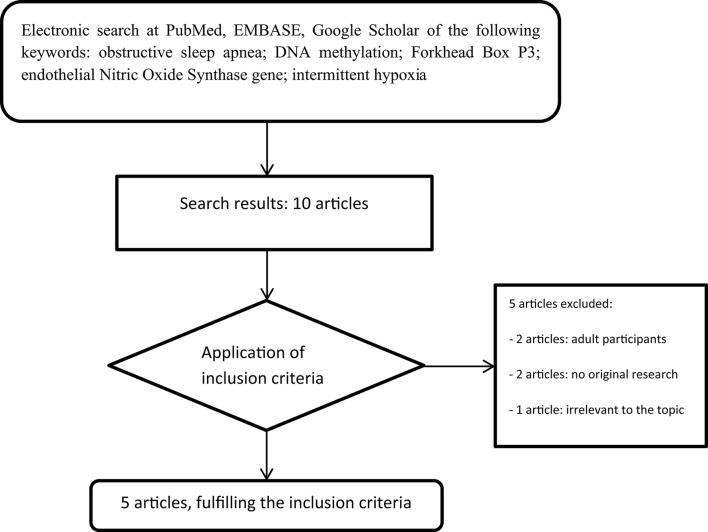
Flow chart of the search strategy.

A comprehensive review of the medical literature was achieved utilizing the US National Library of Medicine's PubMed database, EMBASE, and Google Scholar, published until January 2017. The keywords applied were the following: obstructive sleep apnea AND DNA methylation AND Forkhead Box P3 OR endothelial Nitric Oxide Synthase gene OR intermittent hypoxia. All data were obtained using the given combinations of the aforementioned keywords. The search terms were applied to titles, abstracts, and full texts of the articles to choose the ones fulfill the inclusion criteria. Only studies written in English were included. The search strategy was limited to humans aged 0–18 years and animals. As we previously mentioned, literature is scarce in the nascent field of epigenetics in OSA. A total of 10 studies were initially considered for the final evaluation, references from the chosen articles also screened for relevant studies; and 5 were finally included in the present review. Exclusion criteria were the following: included participants older than 18 years (*n* = 2 articles), not original research (*n* = 2 articles), and not relevant data, specifically one study refers to DNA Methylation in Obesity Hypoventilation Syndrome (*n* = 1 article). Regarding original research, only 2 studies in pediatric population and 3 animal studies derived from literature search. Pertaining to human studies, population differences, including sex differences could not derived due to the tiny samples size and due to the fact that they were conducted from the same institution. With regard to the animal studies there were not any strain/species differences; since one study was performed on neonatal rodents, one on adult, male rodents, and the other one on xenografted mice. Animal models are commonly used in medical research, although interspecies differences should be kept in mind. The followed strategy and search results are presented in Figure 1.

## Results

The systematic literature search yielded a total of 10 records (Figure 1). After screening of titles, abstracts, and full text version, 5 articles were excluded because of other age target groups, review articles, and irrelevant publications. Finally, 5 publications met the criteria for analysis, comprising 161 children aged 4–12 years and adult as well as neonatal rats (16–20). The main characteristics of the included studies are summarized in **Table 3**.

### Forkhead Box P3

Kim and colleagues examined, for the first time, the hypothesis that epigenetic alterations in inflammatory genes may be related with the variation of inflammatory phenotypes in pediatric OSA (16). This was a preliminary study that found an association between Forkhead Box P3 (FOXP3) DNA methylation and high sensitivity CRP (hsCRP) OSA phenotype. The study compared 31 hsCRP OSA patients to 16 low CRP OSA patients and 31 control subjects. By applying a candidate approach authors investigated DNA methylation patterns in 24 major inflammatory genes known to be deregulated in a relatively large cohort of children with OSA (*n* = 78).

They identified a strong association of highly methylated inflammatory genes with elevated CRP levels. However, log FOXP3 DNA methylation patterns, log Myeloid-Related Protein 8/14 (MRP 8/14) levels, and log hsCRP levels demonstrated important group dissimilarities (Table 1). In particular, FOXP3 DNA methylation levels were closely correlated with CRP levels, indicating a potential mechanistic link. Similar associations were also observed with other markers of disease severity including AHI suggesting that epigenetic marks could be used as reliable biomarkers of disease progression, and conceivably epigenetic alterations may be therapeutically adjusted in order to provide epigenetic treatments.

**Table 1:** Characteristics of children with obstructive sleep apnea syndrome and matched control subjects.

	**OSA with high** **hsCRP (*n* = 31)**	**OSA with low hsCRP (*n* = 16)**	**Controls** **(*n* = 31)**
Log MRP 8/14	1.42 ± 0.69 μg/ml	0.69 ± 0.40 μg/ml	0.81 ± 0.67 μg/ml
Log hsCRP	1.90 ± 1.48 mg/dl	0.31 ± 0.03 mg/dl	0.58 ± 0.66 mg/dl
Log FOXP3 DNA methylation	24.2 ± 12.1%	15.5 ± 9.5%	15.9 ± 8.6%

*Modified by Kim et al. (16)*.

*FOXP3, Forkhead Box P3; hsCRP, high-sensitivity C-Reactive Protein; MRP, Myeloid-Related Protein; OSA, Obstructive Sleep Apnea*.

### Endothelial nitric oxide synthase

Kheirandish-Gozal and co-workers speculated that the presence of vascular dysfunction in children with OSA might be linked to epigenetic alterations in the endothelial Nitric Oxide Synthase (eNOS) gene (17). In this study, 35 children were the healthy control group and 36 children had OSA, between them 11 had delays in post-occlusive hyperemic responses (OSAab) as indicated by a significant augmentation in the time needed to attain peak reperfusion flow (Tmax). Children with OSA had higher diastolic blood pressure and serum lipids in morning awakening in comparison with the control subject (Table 2). Using pyrosequencing analyses, authors detected a hyper-methylated CpG position in the core promoter region of the eNOS gene in the OSAab group. This epigenetic mark was linked to children who exhibited impaired eNOS expression combined with deregulated microvascular responses. The study interestingly did not find association of eNOS methylation with AHI severity.

**Table 2:** Characteristics of children with obstructive sleep apnea syndrome with and without endothelial dysfunction and characteristics of control subjects.

	**OSAn (*n* = 25)**	**OSAab (*n* = 11)**	**Controls (*n* = 35)**
Systolic blood pressure, mm Hg	109.2 ± 8.2	108.8 ± 10.7	103.4 ± 7.6
Diastolic blood pressure, mm Hg	64.7 ± 8.1	65.2 ± 7.5	60.9 ± 6.2
Total cholesterol, mg/dL	174.9 ± 36.1	178.3 ± 37.8	159.4 ± 19.3
HDL cholesterol, mg/dL	48.2 ± 13.5	47.4 ± 14.9	54.0 ± 10.3
LDL cholesterol, mg/dL	114.1 ± 36.2	118.5 ± 22.9	93.8 ± 19.1
Triglycerides, mg/dL	106.2 ± 43.5	102.5 ± 45.7	82.7 ± 31.3
Peak hyperaemic response, sec	30.1 ± 8.3	66.7 ± 8.8	27.8 ± 6.1

*Modified by Kim et al. (16)*.

*HDL, High-Density Lipoprotein; LDL, Low-Density Lipoprotein; OSAab, Obstructive Sleep Apnea with delays in postocclusive hyperemic response; OSAn, Obstructive Sleep Apnea with normal hyperemic response*.

### Intermittent hypoxia (Table 3)

Nanduri and colleagues showed that Sprague-Dawley rats of both sexes and adult, male Sprague-Dawley rats exposed to Intermittent Hypoxia (IH), at the neonatal period as well as directly during adulthood, exhibit enhanced hypoxic sensitivity, leading to abnormal breathing (18, 19). With an elegant series of experiments they demonstrated that IH caused elevation of oxidative stress markers in both carotid body and adrenal medulla (18). Further analysis revealed that IH was conducive to hypermethylation of Sod2, a key regulatory enzyme of mitochondrial metabolism. Treatment of rats with hypomethylating agents (decitabine) reversed this phenomenon (18). The formerly outcomes link oxygen homeostasis impairment, throughout neonatal period, with oxidative stress, during adulthood, via mechanisms that involve epigenetic modifications of cellular bioenergetics regulators. Human relevance of the findings were provided in a follow-up study (19) where long term IH was associated with hypertension, spontaneous apneas, heightened carotid body chemosensory reflex, and elevated reactive oxygen species levels in both the carotid body and adrenal medulla. These outcomes were reversible after room air recovery from short term IH, but not from long term IH. Analysis of the molecular mechanisms revealed increased DNA methylation of genes encoding anti-oxidant enzymes and treatment with decitabine reversed these changes. These findings support the notion that irreversible cardiorespiratory abnormalities due to prolonged IH are in part mediated by epigenetic re-programming of the redox state in the carotid body chemosensory reflex pathway.

**Table 3:** Studies assessing genetic alterations in pediatric obstructive sleep apnea syndrome.

**Study**	**Sample size (*n*)**	**Genetic analysis**	**Main results**
Kim et al. (16)	– First phase: 12 children with OSA and HhsCRP or LhsCRP – Second phase: 47 children with OSA and 31 controls	24 inflammatory-related genes evaluated for DNA methylation levels	– Highly methylated inflammatory genes in HhsCRP group – Higher log FOXP3 DNA methylation levels in HhsCRP group than in LhsCRP group – FOXP3 DNA hypermethylation increased in relation to OSA severity
Kheirandish-Gozal et al. (17)	36 prepubertal children, with polysomnographically confirmed OSAn (*n* = 25) or OSAab (*n* = 11) and 35 children in the control group	Blood genomic DNA analyzed for epigenetic changes in the core promoter region of eNOS gene	– Hypermethylated CpG in core promoter region of eNOS gene in OSAab group – eNOS mRNA levels significantly decreased in OSAab group vs. OSAn group
Nanduri et al. (18)	– Rats exposed to IH, i.e., alternating cycles of 5% O_2_ for 15 s and 21% O_2_ for 5 min, 8 h per day for 10 days – Rats in the control group were exposed to rotating cycles of room air	– DNMT mRNA levels in AM and CB and corresponding proteins were analyzed – Analysis of DNA methylation status of CpG islands in rat Sod2 and Duox2 genes	– Rats exposed to IH had elevated levels of DNMT1, DNMT3b mRNA, protein, and significantly elevated oxidative stress (blocked by decitabine) – DNA methylation of Sod2 gene incremented 6- and 12-fold in CB and AM, respectively, in rats exposed to IH – DNA methylation of Duox2 gene was unchanged
Nanduri et al. (19)	– Rats exposed to IH, i.e., alternating cycles of 5% O_2_ for 15 s and 5 min of room air for 10 days (ST-IH) or 30 days (LT-IH) – Rats in the control group were exposed to alternating cycles of room air	– AOE genes were analyzed (Sod1, Sod2, Cat, Txnrd2, Txnrd4, Gpx2) – Analysis of DNA methylation in AM tissues of ST-IH and LT-IH after room air recovery	– AOE mRNA levels in CB and AM of ST-IH group were nearly the same with controls, after 10 days of recovery – AOE genes were reduced in CBs of LT-IH rodents after 30 days of recovery – AM displayed reduced expression of Sod1, Sod 2, Txnrd2, and Prdx4 vs. controls – ST-IH no changes in DNA methylation of AOE genes – LT-IH increased DNA methylation of Sod1, Sod2, Txnrd2, Prdx4 after 30 days of recovery
Cortese et al. (20)	– Mice exposed to chronic IH (XenoIH), i.e., alternating cycles of 90 s 6% O_2_ followed by 21% O_2_ for 12 h and the remaining 12 h of nighttime, O_2_ concentrations were kept at 21% – Mice in the control group (XenoRA) were subjected to continuous circulating 21% O_2_	cirDNA analysis	– A significant increase on plasma cirDNA quantity in XenoIH group – Tumor size and weight in XenoIH were vs. XenoRA group – Mice with invasive tumors had significantly higher plasma cirDNA concentrations than those with non-invasive tumors

*AM, Adrenal Medulla; AOE, Anti-Oxidant Enzyme; Cat, Catalase; CB, Carotid Body; DNMT, DNA methyltransferase; Duox, dual oxidase; eNOS, endothelial nitric oxide synthase; FOXP3, Forkhead Box P3; Gpx, Glutathione peroxidase; HhsCRP, High levels of high-sensitivity C-Reactive Protein; IH, Intermittent Hypoxia; LhsCRP, Low levels of high-sensitivity C-Reactive Protein; LT-IH, Long Term-Intermittent Hypoxia; O_2_, Oxygen; OSA, Obstructive Sleep Apnea; OSAab, Obstructive Sleep Apnea with delays in postocclusive hyperemic response; OSAn, Obstructive Sleep Apnea with normal hyperemic response; Prdx, Peroxiredoxin; Sod, Superoxide dismutase; ST-IH, Short Term-Intermittent Hypoxia; Txnrd, Thioredoxin reductase*.

Another important finding about IH, the hallmark characteristic of OSA, came from the study of Cortese et al. (20). They have demonstrated that IH during sleep, imitating those met in moderately severe patients with OSA, are caused by increases in the exportation of cirDNA into circulation, in tumor and non-tumor-injected mice. In addition, they showed that cirDNA transfers epigenetic alterations that may characterize certain cell subtypes. Finally, they concluded that the intrinsic variability of cirDNA methylation within the tumor may subserve some tumor cell populations to unchain their DNA upon IH exhibitions.

## Discussion

Pediatric OSA is a highly prevalent public health disease entity; however its diagnosis requires labor-intensive steps and is time-consuming. OSA is associated with increased risk for end-organ substantial morbidities, ultimately leading to reduced quality of life. The risk of end-organ morbidities has instigated the search for biomarker signatures and finally the implementation of biomarker-based algorithms for diagnosis and risk assessment (10, 21). Additionally, the previously aforementioned findings have opened the door to a series of researches mandatory to elucidate the extended role of epigenetics in children with underlying OSA. The dichotomous changes in OSA phenotypes boosted the exploration of mechanisms by which such modifications take place.

The transcription factor FOXP3 plays a significant role in immune homeostasis and is the master regulator of T regulatory lymphocytes (Tregs) differentiation, development and function. Kim and co-workers concluded that the numerical impairment of Tregs in children with OSA could be attributed to FOXP3 hypermethylation, thus inducing an imbalance of Th1/Th2 cytokines (16). Intriguingly, OSA appears to shift Th1/Th2 balance toward Th1 (16, 22). Accordingly, methylation of CpG residues suppresses FOXP3 expression, while total demethylation is needed for stable FOXP3 expression (23). Current evidence delineates the pivotal role of epigenetic marks in the induction and stabilization of FOXP3 expression, suggesting that targeted interference with the underlying chromatin remodeling mechanisms can enable development of putatively fruitful future therapeutic applications, in terms of personalized medicine approaches, in numerous diseases.

Whilst, in the study of Kim et al. (16) the groups were not matched for their BMIs and had significantly more obese children in the hsCRP OSA group; which raises the question if the differences in the inflammatory markers are related to obesity or not. The authors have shown, using a multi-regression model, that differences in methylation pattern sustain when matched for the BMI, but the strength of the association is reduced.

Taken together, current literature suggests that OSA provides incremental cardiovascular morbidity risk independent from other confounding factors. Increased hsCRP levels have been reported as a biomarker of disease severity and treatment response (24–26). Nevertheless, data arising from other studies report conflicting results. Epigenetic modifications including lifestyle and other environmental conditions may explain these discrepancies (24, 27, 28).

Pediatric OSA is a risk factor for the occurrence of systemic hypertension and cardiovascular disease. Altered endothelial function represents an early risk marker of cardiovascular morbidity (17, 29–31). Early prediction of endothelial dysfunction in children with OSA is limited by genetic polymorphisms among nitric oxide synthase and endothelin signaling pathways (32–34). Recent studies have shown that epigenetic modifiers, mainly hypermethylation of CpG islands in the promoter region of eNOS, could account for decreased eNOS activity and consequently peripheral vascular impairment in pediatric OSA patients.

Authors of both the aforementioned studies, Kim et al. (16) and Kheirandish-Gozal et al. (17), have stated that is unclear whether the methylation patterns are a cause or an effect of the inflammatory phenotypes of OSA, thus currently only as association has been found. Further studies are needed to determine the relationship and its use either in treatment or as a biomarker. Epigenome-wide multicenter profiling studies are sorely required to delineate end-organ susceptibility in pediatric OSA, offering reliable disease prognosticators and providing new inroads into epigenetic treatments. Our awareness about identification of molecular endotypes is growing through in depth understanding of genotype-phenotype interactions, aiming the transition to precision sleep medicine epoch.

The studies of Nanduri and colleagues are of great clinical relevance, since adult individuals born preterm show increased frequency of high blood pressure and Sleep Disordered Breathing (SDB), indicating that apnea of prematurity predetermines dysfunctions of autonomic system (18, 19). The extracted data suggest that the premature onset of autonomic dysfunction in adult individuals born preterm can exist due to exaggerated hypoxic sensing caused by epigenetic transgenerational modifications.

Cortese et al. found that exposure to IH during sleep was related to heightened plasma cirDNA in xenografted and control mice; appealing, similar findings concur with reports of elevated plasma cirDNA amount in OSA patients (20). Even though plasma cirDNA has been widely reported in the majority of cancer types, its implementation as biomarker has been doubted due to its inter-patient variation (20). It is worth noting that recent studies, using murine models of OSA being selectively exposed to IH (35, 36) or sleep fragmentation (37, 38), have shown, unanimously, increased tumor growth, migration, cell proliferation, invasiveness, and adverse metabolic phenotype in adult offspring.

In an effort to develop more convenient, child-friendly screening methods, few studies analyzed proteins in urine, which is an extremely easy obtainable material, and evaluate if urinary proteome reveals clusters that are differentially expressed in the urine of polysomnography-confirmed OSA children (39–41). In one of the initial pilot studies, more than one decade ago, the authors stratified 11 children with OSA and 11 control children who underwent overnight sleep studies followed by a first-morning urine sample and, subsequently, a proteomic analysis of urine samples took place (38). A unique map of proteins was generated; increased expression of gelsolin and perlecan in the OSA group suggested that the OSA-related episodic hypoxia may induce alterations in protein permeability or increased catabolism of these proteins and, accordingly, their excretion in urine.

The quest of other alternative samples, such as exhaled breath and saliva may arise (10). Those preliminary proteomic assessments disclose that pediatric OSA is correlated with specific and consistent changes in urinary concentrations of certain protein clusters. The nascent idea of a urine box able to talk the story about a child suffering or not from OSA appears vastly attractive and accomplishable (41).

Our study has some major limitations that they must be considered in order to allow interpretation of the described findings. Firstly, studies are apparently lacking in the field of DNA methylation in pediatric OSA leading to a broadly underrated disease pathogenesis and phenotyping. Secondly, the implementation of the findings exported from animal models it is not always successful in humans. Thirdly, the lack of large sample sizes and samples from all over the world makes the results not easy to be generalize in both sexes and to populations outside of the samples geographical areas.

Conclusively, there is a preliminary but emerging body of evidence suggesting that epigenetic repertoire may represent an important component of phenotypic variation observed in pediatric sleep disorders. There are other studies which look into epigenetic modifications other than DNA methylation, for example Karla et al. found an association of a single nucleotide polymorphisms (SNPs) in the region of ApoE with OSA status in children (42); Gozal et al. published a link between a SNP in the p22 phox subunit of the NOX gene and cognitive deficits in children with OSA (43); Cortese et al. have shown histone modifications in oxidative stress pathways in aorta macrophages in IH exposed mice (44); Khalyfa et al. found that miRNA-630 was reduced in children with OSA and endoththelial dysfunction (45).

Studies to explore the potential association of DNA methylation patterns with the disease severity on adult population with OSA are starting to emerge (46). Notwithstanding, there are extensive deficits in our perception about mechanisms governing OSA and consequences. Future bioinformatic analyses including genome-wide association approaches based on microarrays or next-generation sequencing are required to clarify epigenomic profiles related with certain phenotypes in pediatric as well as adult sleep disorders (28). Using epigenetics is a major challenge and will offer the reward of implementing this knowledge to the development of novel diagnostic biomarkers and targeted therapeutic tools to genetically predisposed individuals.

## Author contributions

PS, EVP, EN, and AT collected and analyzed the data, drafted the initial manuscript, revised the manuscript, and approved the final manuscript as submitted. MK drafted the initial manuscript and approved the final manuscript as submitted. EMP analyzed the data, supervised drafting of the initial manuscript, supervised revision of the manuscript, and approved the final manuscript as submitted.

### Conflict of interest statement

The authors declare that the research was conducted in the absence of any commercial or financial relationships that could be construed as a potential conflict of interest.

## References

[1] Tan HL Gozal D Kheirandish-Gozal L. Obstructive sleep apnea in children: a critical update. Nat Sci Sleep (2013) 5:109–23. 10.2147/NSS.S51907 24109201 PMC3792928

[2] Gozal D. Sleep, sleep disorders and inflammation in children. Sleep Med. (2009) **10**(Suppl. 1):S12–6. 10.1016/j.sleep.2009.07.003 19647481

[3] Jaenisch R Bird A. Epigenetic regulation of gene expression: how the genome integrates intrinsic and environmental signals. Nat Genet. (2003) **33**(Suppl.):245–54. 10.1038/ng1089 12610534

[4] Wolffe AP Matzke MA. Epigenetics: regulation through repression. Science (1999) 286:481–86. 10.1126/science.286.5439.481 10521337

[5] Urnov FD Wolffe AP. Above and within the genome: epigenetics past and present. J Mammary Gland Biol Neoplasia (2001) 6:153–67. 10.1023/A:1011304606604 11501576

[6] Ren J Singh BN Huang Q Li Z Gao Y Mishra P et al. DNA hypermethylation as a chemotherapy target. Cell Signal. (2011) 23:1082–93. 10.1016/j.cellsig.2011.02.003 21345368

[7] Yang IV Schwartz DA. Epigenetic control of gene expression in the lung. Am J Respir Crit Care Med. (2011) 183:1295–301. 10.1164/rccm.201010-1579PP 21596832 PMC3114059

[8] Durham AL Wiegman C Adcock IM. Epigenetics of asthma. Biochim Biophys Acta (2011) 1810:1103–9. 10.1016/j.bbagen.2011.03.006 21397662

[9] Kheirandish-Gozal L Gozal D. Genotype-phenotype interactions in pediatric obstructive sleep apnea. Respir Physiol Neurobiol. (2013) 189:338–43. 10.1016/j.resp.2013.03.016 23563156 PMC3751986

[10] Khalyfa A Gileles-Hillel A Gozal D. The challenges of precision medicine in obstructive sleep Apnea. Sleep Med Clin. (2016) 11:213–26. 10.1016/j.jsmc.2016.01.003 27236058

[11] Cortese R Khalyfa A Bao R Andrade J Gozal D. Epigenomic profiling in visceral white adipose tissue of offspring of mice exposed to late gestational sleep fragmentation. Int J Obes. (2015) 39:1135–42. 10.1038/ijo.2015.38 25801690 PMC4496299

[12] Khalyfa A Carreras A Almendros I Hakim F Gozal D. Sex dimorphism in late gestational sleep fragmentation and metabolic dysfunction in offspring mice. Sleep (2015) 38:545–57. 10.5665/sleep.4568 25325475 PMC4355894

[13] Khalyfa A Mutskov V Carreras A Khalyfa AA Hakim F Gozal D. Sleep fragmentation during late gestation induces metabolic perturbations and epigenetic changes in adiponectin gene expression in male adult offspring mice. Diabetes (2014) 63:3230–41. 10.2337/db14-0202 24812424 PMC4171662

[14] Mutskov V Khalyfa A Wang Y Carreras A Nobrega MA Gozal D. Early-life physical activity reverses metabolic and Foxo1 epigenetic misregulation induced by gestational sleep disturbance. Am J Physiol Regul Integr Comp Physiol. (2015) 308:R419–30. 10.1152/ajpregu.00426.2014 25568076 PMC4346758

[15] Groom A Elliott HR Embleton ND Relton CL. Epigenetics and child health: basic principles. Arch Dis Child. (2011) 96:863–9. 10.1136/adc.2009.165712 20656732

[16] Kim J Bhattacharjee R Khalyfa A Kheirandish-Gozal L Capdevila OS Wang Y et al. DNA methylation in inflammatory genes among children with obstructive sleep apnea. Am J Respir Crit Care Med. (2012) 185:330–8. 10.1164/rccm.201106-1026OC 22077067 PMC3297110

[17] Kheirandish-Gozal L Khalyfa A Gozal D Bhattacharjee R Wang Y. Endothelial Dysfunction in children with Obstructive Sleep Apnea is associated with epigenetic changes in the eNOS gene. Chest (2013) 143:971–7. 10.1378/chest.12-2026 23328840 PMC3616686

[18] Nanduri J Makarenko V Reddy VD Yuan G Pawar A Wang N et al. Epigenetic regulation of hypoxic sensing disrupts cardiorespiratory homeostasis. Proc Natl Acad Sci USA. (2012) 109:2515–20. 10.1073/pnas.1120600109 22232674 PMC3289330

[19] Nanduri J Peng YJ Wang N Khan SA Semenza GL Kumar GK et al. Epigenetic regulation of redox state mediates persistent cardiorespiratory abnormalities after long-term intermittent hypoxia. J Physiol. (2017) 595:63–77. 10.1113/JP272346 27506145 PMC5199741

[20] Cortese R Almendros I Wang Y Gozal D. Tumor circulating DNA profiling in xenografted mice exposed to intermittent hypoxia. Oncotarget (2015) 6:556–69. 10.18632/oncotarget.2785 25415227 PMC4381615

[21] Kheirandish-Gozal L Gozal D. Pediatric OSAS morbidity biomarkers: the hunt is finally on! Chest (2017) 151:500–6. 10.1016/j.chest.2016.09.026 27720883 PMC5310114

[22] Yang IV. Sleep, immunology, and epigenetics: tip of an iceberg. Am J Respir Crit Care Med. (2012) 185:243–5. 10.1164/rccm.201111-2028ED 22298364

[23] Liu J Lluis A Illi S Layland L Olek S von Mutius E. T regulatory cells in cord blood–foxp3 demethylation as reliable quantitative marker. PLoS ONE (2010) **5**:e13267. 10.1371/journal.pone.0013267 20967272 PMC2953505

[24] Yokoe T Minoguchi K Matsuo H Oda N Minoguchi H Yoshino G et al. Elevated levels of c-reactive protein and interleukin-6 in patients with obstructive sleep apnea syndrome are decreased by nasal continuous positive airway pressure. Circulation (2003) 107:1129–34. 10.1161/01.CIR.0000052627.99976.18 12615790

[25] Li AM Chan MH Yin J So HK Ng SK Chan IH et al. C-reactive protein in children with obstructive sleep apnea and the effects of treatment. Pediatr Pulmonol. (2008) 43:34–40. 10.1002/ppul.20732 18041751

[26] Kheirandish-Gozal L Capdevila OS Tauman R et al. Plasma c-reactive protein in nonobese children with obstructive sleep apnea before and after adenotonsillectomy. J Clin Sleep Med. (2006) 2:301–4. 17410279 PMC1847566

[27] Kaditis AG Alexopoulos EI Kalampouka E Kostadima E Germenis A Zintzaras E et al. Morning levels of c-reactive protein in children with obstructive sleep-disordered breathing. Am J Respir Crit Care Med. (2005) 171:282–6. 10.1164/rccm.200407-928OC 15557130

[28] Guilleminault C Kirisoglu C Ohayon MM. C-reactive protein and sleep-disordered breathing. Sleep (2004) 27:1507–11. 10.1093/sleep/27.8.1507 15683141

[29] Somers VK White DP Amin R Abraham WT Costa F Culebras A et al. Sleep apnea and cardiovascular disease: an American Heart Association/American College of Cardiology Foundation scientific statement from the American Heart Association Council for High Blood Pressure Research Professional Education Committee, Council on Clinical Cardiology, Stroke Council, and Council on Cardiovascular Nursing. In Collaboration with the National Heart, Lung, and Blood Institute National Center on Sleep Disorders Research (National Institutes of Health). Circulation (2008) 118:1080–111. 10.1161/CIRCULATIONAHA.107.189420 18725495

[30] Kato M Roberts-Thomson P Phillips BG Haynes WG Winnicki M Accurso V et al. Impairment of endothelium-dependent vasodilation of resistance vessels in patients with obstructive sleep apnea. Circulation (2000) 102:2607–10. 10.1161/01.CIR.102.21.2607 11085964

[31] Gozal D Kheirandish-Gozal L Serpero LD Sans Capdevila O Dayyat E. Obstructive sleep apnea and endothelial function in school-aged nonobese children: effect of adenotonsillectomy. Circulation (2007) 116:2307–14. 10.1161/CIRCULATIONAHA.107.696823 17967978

[32] Bhattacharjee R Kim J Alotaibi WH Kheirandish-Gozal L Capdevila OS Gozal D. Endothelial dysfunction in children without hypertension: potential contributions of obesity and obstructive sleep apnea. Chest (2012) 141:682–91. 10.1378/chest.11-1777 22030801 PMC3296460

[33] Chatsuriyawong S Gozal D Kheirandish-Gozal L Bhattacharjee R Khalyfa AA Wang Y et al. Polymorphisms in nitric oxide synthase and endothelin genes among children with obstructive sleep apnea. BMC Med Genomics (2013) **6**:29. 10.1186/1755-8794-6-29 24010499 PMC3844410

[34] Chatsuriyawong S Gozal D Kheirandish-Gozal L Bhattacharjee R Khalyfa AA Wang Y et al. Genetic variance in nitric oxide synthase and endothelin genes among children with and without endothelial dysfunction. J Transl Med. (2013) **11**:227. 10.1186/1479-5876-11-227 24063765 PMC3849009

[35] Almendros I Wang Y Becker L Lennon FE Zheng J Coats JR et al. Intermittent hypoxia-induced changes in tumor-associated macrophages and tumor malignancy in a mouse model of sleep apnea. Am J Respir Crit Care Med. (2014) 189:593–601. 10.1164/rccm.201310-1830OC 24471484 PMC3977714

[36] Hakim F Wang Y Zhang SX Zheng J Yolcu ES Carreras A et al. Fragmented sleep accelerates tumor growth and progression through recruitment of tumor-associated macrophages and TLR4 signaling. Cancer Res. (2014) 74:1329–37. 10.1158/0008-5472.CAN-13-3014 24448240 PMC4247537

[37] Trzepizur W Khalyfa A Qiao Z Popko B Gozal D. Integrated stress response activation by sleep fragmentation during late gestation in mice leads to emergence of adverse metabolic phenotype in offspring. Metabolism (2017) 69:188–98. 10.1016/j.metabol.2017.01.026 28139216

[38] Krishna J Shah ZA Merchant M Klein JB Gozal D. Urinary protein expression patterns in children with sleep-disordered breathing: preliminary findings. Sleep Med. (2006) **7:**221–7. 10.1016/j.sleep.2005.09.010 16564219

[39] Gozal D Jortani S Snow AB Kheirandish-Gozal L Bhattacharjee R Kim J et al. Two-dimensional differential in-gel electrophoresis proteomic approaches reveal urine candidate biomarkers in pediatric obstructive sleep apnea. Am J Respir Crit Care Med. (2009) 180:1253–61. 10.1164/rccm.200905-0765OC 19797158 PMC2796735

[40] Kheirandish-Gozal L McManus CJ Kellermann GH Samiei A Gozal D. Urinary neurotransmitters are selectively altered in children with obstructive sleep apnea and predict cognitive morbidity. Chest (2013) 143:1576–83. 10.1378/chest.12-2606 23306904 PMC3673660

[41] Tan HL Kheirandish-Gozal L Gozal D. The promise of translational and personalised approaches for paediatric obstructive sleep apnoea: an “Omics” perspective. Thorax (2014) 69:474–80. 10.1136/thoraxjnl-2013-204640 24550060

[42] Kalra M Pal P Kaushal R Amin RS Dolan LM Fitz K et al. Association of ApoE genetic variants with obstructive sleep apnea in children. Sleep Med. (2008) 9:260–65. 10.1016/j.sleep.2007.05.001 17658295

[43] Gozal D Khalyfa A Capdevila OS Kheirandish-Gozal L Khalyfa AA Kim J. Cognitive function in prepubertal children with obstructive sleep apnea: a modifying role for NADPH oxidase p22 subunit gene polymorphisms? Antioxid Redox Signal. (2012) 16:171–77. 10.1089/ars.2011.4189 21902598 PMC3250922

[44] Cortese R Gileles-Hillel A Khalyfa A Almendros I Akbarpour M Khalyfa AA et al. Aorta macrophage inflammatory and epigenetic changes in a murine model of obstructive sleep apnea: potential role of CD36. Sci Rep. (2017) **7**:43648. 10.1038/srep43648 28240319 PMC5327416

[45] Khalyfa A Kheirandish-Gozal L Khalyfa AA Philby MF Alonso-Álvarez ML Mohammadi M et al. Circulating plasma extracellular microvesicle MicroRNA cargo and endothelial dysfunction in children with obstructive sleep apnea. Am J Respir Crit Care Med. (2016) 194:1116–26. 10.1164/rccm.201602-0323OC 27163713 PMC5114451

[46] Chen YC Chen TW Su MC Chen CJ Chen KD Liou CW et al. Whole genome DNA methylation analysis of obstructive sleep apnea: IL1R2, NPR2, AR, SP140 methylation and clinical phenotype. Sleep (2016) 39:743–55. 10.5665/sleep.5620 26888452 PMC4791608

